# Development of a Virosomal RSV Vaccine Containing 3D-PHAD® Adjuvant: Formulation, Composition, and Long-Term Stability

**DOI:** 10.1007/s11095-018-2453-y

**Published:** 2018-07-03

**Authors:** J. Lederhofer, J. van Lent, F. Bhoelan, Z. Karneva, A. de Haan, J.C. Wilschut, T. Stegmann

**Affiliations:** 1 University Medical Centre Groningen, Department of Medical Microbiology, University of Groningen, Groningen, The Netherlands; 2 Laboratory of Virology, Wageningen University, Wageningen, The Netherlands; 3 Mymetics BV, Leiden, The Netherlands

**Keywords:** adjuvant, monophosphoryl lipid A, respiratory syncytial virus, single particle tracking, vaccine, virosomes

## Abstract

**Purpose:**

Characterization of virosomes, in late stage preclinical development as vaccines for Respiratory Syncytial Virus (RSV), with a membrane-incorporated synthetic monophosphoryl lipid A, 3D-PHAD® adjuvant.

**Methods:**

Virosomes were initially formed by contacting a lipid film containing 3D-PHAD® with viral membranes solubilized with the short chain phospholipid DCPC, followed by dialysis, later by adding solubilized 3D-PHAD to viral membranes, or to preformed virosomes from DMSO.

**Results:**

Virosomes formed from lipid films contained the membrane glycoproteins G and F, at similar F to G ratios but lower concentrations than in virus, and the added lipids, but only a fraction of the 3D-PHAD®. By single particle tracking (SPT), the virosome size distribution resembled that seen by cryo-electron microscopy, but dynamic light scattering showed much larger particles. These differences were caused by small virosome aggregates. Measured by SPT, virosomes were stable for 300 days. 3DPHAD ® incorporation in virosomes could be enhanced by providing the adjuvant from DCPC solubilized stock, but also by adding DMSO dissolved adjuvant to pre-formed virosomes. Virosomes with 0.1 mg/mg of 3D-PHAD®/viral protein from DMSO induced antibody titers similar to those by virosomes containing 0.2 mg/mg of DCPC-solubilized 3D-PHAD®.

**Conclusions:**

Stable 3D-PHAD® adjuvanted RSV virosomes can be formulated.

## Introduction

Respiratory Syncytial Virus (RSV) is a major cause of viral bronchiolitis among infants and young children, and also causes serious disease in immunocompromised individuals and the elderly. Worldwide, approximately 120 million people are affected by a severe acute respiratory infection (SARI) each year, among whom 1 million patients do not survive the infection (1,2). It has been shown that RSV is one of the most common pathogens to cause SARI (3). Despite the impact of RSV on global health, there is no vaccine available for prevention of RSV infection. This is in part due to the disastrous outcome of an early clinical study among young children in the 1960s. In this trial, a formalin-inactivated RSV vaccine (FI-RSV) induced enhanced respiratory disease in vaccinees upon natural infection, leading to increased morbidity and the death of two children (4–7). This unexpected response was found to be due in part to the induction by the FI-RSV vaccine of low-affinity and poorly neutralizing antibodies directed to the fusion (F) glycoprotein of RSV (8–13).

While RSV vaccine development has been delayed, an effective RSV vaccine remains urgently needed. At the same time, there is an increasing awareness that different vaccine formulations may be required for different target groups for vaccination. Live-attenuated vaccines, which mimic exposure to wild-type RSV, aim at protection of infants and young children, while avoiding enhanced respiratory disease. Alternatively, young infants in the first critical months after birth may be protected through vaccination of the mothers during the third trimester of pregnancy (14). In this case, the main aim of vaccination is a short-term induction of high titers of virus-neutralizing (VN) antibodies for efficient transfer through the placenta. An inactivated vaccine comprising at least the RSV F glycoprotein may well be optimally suited for this purpose. Finally, an RSV vaccine is also needed for the elderly and immunocompromised individuals. Here, it is a gradual senescence or dysfunction of the immune system that may cause symptomatic disease after RSV infection, despite repeated exposure to the virus earlier on in life. Clearly, here the aim of vaccination is to boost the pre-existing immune response. A particle-based formulation comprising both the F and G glycoproteins of the virus and containing a powerful adjuvant, would appear to be optimal (8,15–18).

We are developing a virosomal vaccine targeting the elderly and pregnant women. Virosomes are reconstituted viral envelopes that contain the membrane glycoproteins of the virus but lack the viral nucleocapsid. Properly produced virosomes retain the receptor-binding and membrane fusion characteristics of the virus from which they are derived, as has been shown extensively for influenza virosomes, (19–21), indicating that the native structure of the membrane proteins was preserved. For RSV, this could be crucial, since with the early FI-RSV vaccine, discussed above, critical epitopes of the viral F glycoprotein were disrupted, which led to induction of low-affinity antibodies with a deficient capacity to neutralize the virus (22,23). It has been shown that potently neutralizing antibodies directed against the F glycoprotein bind to the native conformation of the (24–26). The second envelope protein, the attachment (G) protein, is the receptor binding protein of the virus (27). Combinations of F and G protein in RSV vaccines have been shown to be substantially more immunogenic than F or G alone (4,28).

In initial RSV virosome vaccine candidates, we tested a lipophilic adjuvant, monophosphoryl lipid A (MPLA) (Kamphuis *et al*., 2012) and a derivative, 3-desacyl MPLA, incorporated in the virosomal membrane (29). 3-desacyl MPLA is used as an adjuvant in several marketed human vaccines. 3-desacyl MPLA is 10.000× less toxic than bacterial lipopolysaccharide (LPS), from which it is derived. Nonetheless, it remains a potent activator of the immune system (30). MPLA is known to activate the immune system through engagement of Toll-like-receptor 4 (TLR4) (31). This activation skews the immune response towards a Th1 T-cell response, leading to increased antibody levels of the favorable IgG1 subtype (32). Kamphuis *et al*. have shown that incorporation of MPLA in RSV virosomes results in increased virus-neutralizing antibody responses in mice and cotton rats compared to non-adjuvanted virosomes (33). The experiments of Kamphuis *et al*. also demonstrated protection of mice and cotton rats against viral challenge after immunization with MPLA-containing virosomes (34). Thus, MPLA is a potentially suitable adjuvant for RSV virosome vaccines. However, MPLA and 3-desacyl MPLA are complex mixtures of about 20 molecules, differing in the number and length of acyl chains, for example, complicating vaccine production.

The present study describes biochemical and physical characterization of a virosomal RSV candidate vaccine containing the synthetic adjuvant 3D-PHAD®, identical to one of the most active molecules present in 3-desacyl MPLA. The study provides size distribution analysis by various techniques, including single-particle tracking (SPT), dynamic light scattering (DLS) and cryogenic transmission electron microscopy (cryo-TEM), as stability studies by SPT, as well as a quantitative account of the lipid, adjuvant and protein composition of the virosomes, methods for incorporating the adjuvant, and initial comparative immunogenicity studies.

## Materials and Methods

### Virus and Cell Culture

CCL-81 Vero cells (ATCC, Wesel, Germany) were grown on Cytodex-1 beads (GE Healthcare, Eindhoven, The Netherlands) in 500 mL disposable spinner flasks (100 mm top cap and 2 angled sidearms, Corning, Wiesbaden, Germany) with serum-free culture medium Optipro-SFM supplemented with Pen/Strep and L-Glutamine (Westburg, Leusden, The Netherlands). The cells were infected with RSV strain A2 (American Type Culture Collection, ATCC VR1540), with a multiplicity of infection (MOI) of 0.001, at a nucleus count of 8 × 10^5^ cells/ml.

The virus was harvested at 50–80% of cytopathic effect (CPE). Cytodex-1 beads and cell debris were removed by filtration through a Pall mini Profile filter® capsule with a pore diameter of 10 μm (Pall, Amsterdam, The Netherlands), residual cellular DNA was digested by treatment with benzonase (Novagus, Merck, Schwalbach am Taunus, Germany), and the supernatant was clarified through a filter train combining Sartopure PP2 filters with a pore size of 1.2 and 0.65 μm (Sartorius, Goettingen, Germany) to remove further particle debris. The material was concentrated by tangential flow ultrafiltration using a Midikros 145cm^2^, pore size 0.05 μm, polysulfone (PS) UF/DF filter (Spectrum labs, Breda, The Netherlands) and the medium was exchanged for PBS buffer (137 mM NaCl, 2.7 mM KCl, 10 mM Na_2_HPO_4_, 1.8 mM KH_2_PO_4_, pH 7.4) by diafiltration. The virus was purified from the concentrate by gel filtration (size exclusion) chromatography. The purified and concentrated virus was rapidly frozen with cryoprotectant (10% sucrose (*w*/*v*)) and stored at −80°C until further use.

### Virosome Production

RSV virosome formulation and production were adapted from Stegmann *et al*. (2010)*.* Briefly, purified RSV A2 virus was concentrated by tangential flow ultrafiltration using a 26 cm^2^, molecular weight cut-off (MWCO) 30 kDa PS ultrafiltration hollow-fiber filter (GE Healthcare), the cryprotectant was exchanged for HNE buffer (5 mM Hepes, 145 mM NaCl, 1 mM EDTA, pH 7.4) by diafiltration, and concentrated virus was dissolved in 100 mM 1,2 dihexanoyl-*sn*-glycero-3-phosphocholine (DCPC) (Avanti Polar Lipids, Alabaster, AL, USA) in HNE buffer. The nucleocapsid was removed by ultracentrifugation in a table-top ultracentrifuge, S100 AT4 rotor, at 50 k rpm for 30 min, and the viral supernatant was collected. In a first set of experiments, a mixture of 1,2-dioleoyl-*sn*-glycero-3-phosphatidylethanolamine (DOPE), 1,2-dioleoyl-*sn*-glycero-3-phosphatidylcholine (DOPC) and cholesterol (all from Avanti), also containing 3-deacyl-phosphorylated hexa-acyl disaccharide (3D-PHAD®) (35) (Avanti), in a 2:1 chloroform/methanol solution was evaporated to form a dry lipid film in a glass tube. The film was dissolved in the viral supernatant to attain final concentrations of 850 nmol DOPE, 425 nmol DOPC, 255 nmol cholesterol, and 300 nmol 3D-PHAD® per mg of total viral protein in the supernatant.

Alternatively, stock solutions of lipids were prepared in 200 mM DCPC in HNE and a stock solution of 3D-PHAD® (1 mg/ml) was prepared in 500 mM DCPC in HNE, and these were mixed with the viral supernatant, as further indicated in the Results section. The supernatant/lipid mixtures were incubated for 15 min on ice, filtered through an 0.22 μm cellulose acetate filter (Whatman, Sigma Aldrich, Zwijndrecht, The Netherlands) and dialyzed in a gamma-irradiated slide-A-lyzer cassette (10kD cut-off; Thermo Scientific, Geel, Belgium) against 6 × 2 l of PBS (pH 7.4) and 1 × 2 L of HNE in total for 48 h. After dialysis, virosomes were stored at 4°C until further use.

### Sucrose Density Gradient Analysis

The virosomes were analyzed by equilibrium sucrose density gradient centrifugation on a 10–60% (*w*/*v* linear sucrose gradient in HNE buffer as described earlier (36). Gradients were centrifuged in a Hitachi centrifuge for 60 h in an AH650 rotor at 50 k rpm (296,005 g). Fractions of 0.5 ml were collected from the gradient and analyzed for protein using a Bio-Rad Bradford protein assay (BioRad, Veenendaal, The Netherlands) (37), phospholipid phosphate as described before (38) and density by refractometry.

### Biochemical Analysis of RSV Virosomes

#### Thin Layer Chromatography

The virosomes were analyzed for the presence of incorporated lipids and adjuvant by TLC. TLC plates were activated at 150°C for 30 min before use. Virosome samples (non-extracted), and control samples (dissolved in chloroform/methanol, 2:1) were applied onto the TLC plates with a Hamilton syringe. Control samples were DOPE, DOPC and synthetic 3D-PHAD®, 1 to 1.5 nmol each. The plates were dried and then eluted with chloroform/methanol/water (100:75:15 by vol), and subsequently dried and incubated, under gentle shaking, for 30 s in 15 ml cerium molybdate stain (Hanessian stain: ammonium molybdate, cerium sulfate, and sulfuric acid), and developed for 10 min at 150°C. TLC plates were scanned and the intensity of each spot (DOPE, DOPC and 3D-PHAD®) was semi-quantified by ImageJ.

#### HPLC and LC-MS

To quantify the incorporated amounts of lipid and synthetic 3D-PHAD®, virosome preparations, prepared with pre-dissolved lipids and 3D-PHAD®, were analyzed by TNO Triskelion (Zeist, The Netherlands) by high-performance liquid chromatography (HPLC) for DOPC, DOPE and cholesterol. The HPLC system Thermo Scientific Ultimate 3000 was used for this analysis with a Waters Acquity BEH Phenyl 1.7 μm, 2.1 × 100 mm column, at a column temperature of 40°C and a flow rate of 0.5 ml/min. Mobile phase A contained 0.1% formic acid, 10 mM NH_4_Ac, 5% methanol and water, mobile phase B contained 0.1% formic acid, 10 mM NH_4_Ac in 100% methanol and the injection volume was 1 μl. Liquid chromatography–mass spectrometry (LC-MS) was conducted by M-Scan (Geneva, Switzerland) and or by Avanti Polar Lipids (Alabaster, AL, USA), using proprietary methods, to determine the amount of 3D-PHAD®, incorporated in the virosomal membrane.

#### SDS-Page

Virosomes were analyzed on a SDS-PAGE gel (RunBlue SDS Gel 16%, Westburg, The Netherlands) followed by silver staining according to manufactures protocol (ProteoSilver Silver stain kit, Sigma-Aldrich, Zwijndrecht, The Netherlands). Mark12 (Thermofisher, Breda, The Netherlands) protein standard was taken along to determine the size of each protein.

### Electron Microscopy

#### Immunogold Labeling

For immunogold labeling, Palivizumab a humanized monoclonal antibody against F protein (ASD Specialty Heath Care Inc., Chicago IL) and MAB858–2 (Millipore), a mouse-derived monoclonal antibody against G protein were used. FCF-300-Ni Mesh grids (Electron Microscopy Science, Hatfield, PA, USA) were first incubated, face downward, on a droplet of sample (virosomes 1:10 dilution, virus undiluted), pH 7.4 for 5 min, then blocked with blocking agent (Aurion, Wageningen, The Netherlands) for 30 min and incubated for 1 h with Palivizumab and MAB858–2, at a dilution of 1:100 in PBS (Lonza, Breda, The Netherlands) containing 5% blocking agent (Aurion). After washing in PBS with 5% blocking agent, grids were incubated for 1 h with secondary antibody, 1:20 diluted in PBS/5% blocking agent, then washed in PBS/5% blocking agent and afterwards in PBS. Secondary antibodies were a 6-nm gold-coupled goat anti-human antibody for Palivizumab and a 15-nm gold-coupled goat anti-mouse antibody for MAB858–2. Grids were fixed with 1% glutaraldehyde in PBS for 10 min, followed by washing with water. Grids were stained for 30 s with 1% uranyl acetate and air dried for EM analysis. Samples were also single -labeled to detect either F or G proteins. For this, same protocol was used as described here. Secondary antibodies were a 10-nm gold coupled goat anti-human antibody for Palivizumab and a 6-nm gold coupled goat anti-mouse antibody for MAB858–2. Sample preparation was done in duplicate. For negative controls a random IgG mouse antibody and human-anti-human actin-beta antibody (AbD Serotec, Oxford, UK) were used. Images were recorded with a Veleta camera on a JEOL JEM 1011 transmission electron microscope and analyzed with the software iTEM (Soft Imaging System, Munster, Germany).

#### Cryogenic TEM

Cryogenic transmission electron microscopy (TEM) analyses were performed at the Electron Microscopy Centre of the Wageningen University (The Netherlands). Quantifoil R2/2 holey carbon grids were exposed to a glow discharge in air for 20 s. Four μl of the suspension was applied to each grid, which were then blotted and vitrified in liquid ethane using a Vitrobot (FEI Company). Frozen specimens were observed at −180°C in a JEOL JEM2100 transmission electron microscope (JEOL Ltd., Japan) equipped with a Gatan CT3500 cryoholder and a Gatan US4000 camera. Images were taken at 2–4 μm under focus.

### Single-Particle Tracking

Particle size distribution of the virosomal RSV vaccine was evaluated using a NanoSight LM10 nanosizer with a 60 mW 405 nm laser (NanoSight, Malvern Instruments Ltd., UK) and a Hamamatsu Ocra Flash 2.8 CMOS camera (Hamamatsu Photonics KK, Hamamatsu, Japan). Before and after each sample measurement, the sample cell was cleaned and dried according to instructions of Malvern Instruments. An appropriate dilution of the samples was prepared to obtain an optimal particle concentration between 40 and 100 particles/frame. Samples were diluted in HNE buffer in two or three steps. Seven consecutive 60-s videos were recorded for each dilution. Shutter and gain settings were optimized for each sample. The camera histogram gating was adjusted for each individual measurement to maximize sensitivity. Data analysis was performed in NanoSight NTA 3.0 or NTA 3.1 software (Malvern Instruments) in batch mode, using finite track length adjusted (FTLA) weighting.

### Dynamic Light Scattering (DLS)

The particle size of the virosomes was also determined using dynamic light scattering (DLS) in a Zetasizer Nano-ZS90 (Malvern Instruments). Intensity-size distribution graphs, Z-average (mean cluster size based on the intensity of scattered light) and polydispersity index values were recorded. Each analysis typically comprised 3 consecutive measurements including the Z-average diameter.

### Ethical Statement

Animal experiments were evaluated and approved by the Committee for Animal Experimentation (DEC) of the University Medical Center Groningen, according to the guidelines provided by the Dutch Animal Protection Act (permit number DEC5662). Immunizations were conducted under isoflurane anesthesia, and every effort was made to minimize animal suffering.

### Animals and Immunizations

Female Balb/c mice (OlaHsd, specific pathogen free [SPF]), 6–8 weeks old were supplied by Harlan (Zeist, The Netherlands). Animals were immunized IM, under light isoflurane anesthesia, by injecting 50 μl of the vaccine in the calf muscles of both hind legs (25 μl per leg), at a dose of 5 μg of viral protein per immunization.

Anti-RSV IgG ELISAs and neutralizing antibody tests were performed as described before (33,36) Briefly, for ELISA, 96-well plates were coated overnight with betapropiolactone (BPL)-inactivated RSV. After blocking, SV-coated plates were incubated with two-fold serial dilutions of mouse sera and then incubated with horseradish-peroxidase-coupled goat anti-mouse IgG, (Southern Biotech) for detection of serum IgG antibody levels, stained with o-Phenylenediamine (OPD; Sigma-Aldrich, St Louis, MO, USA), and read in a ELISA plate reader at 492 nm. IgG titers were determined as the reciprocal of the highest dilution with an optical density (OD) reading of at least 0.2, after subtraction of the OD of the blank. The titers of virus neutralizing antibodies in were determined by incubation of serial two-fold dilutions of decomplemented serum with infectious virus, and infecting Hep-2 cells with the mixtures (33).

## Results

### Physical and Biochemical Analysis of the Composition of RSV Virosomes

Virosomes were prepared from purified RSV virus, strain A2, as described in Materials and Methods. Briefly, purified RSV virus was solubilized with DCPC, the viral nucleocapsid was removed by ultracentrifugation, the supernatant was added to a dry lipid film consisting of DOPC, DOPE, cholesterol and 3D-PHAD®, the film was solubilized in the DCPC-containing supernatant and, after sterile filtration, the final mixture was dialyzed.

**Fig. 1:**
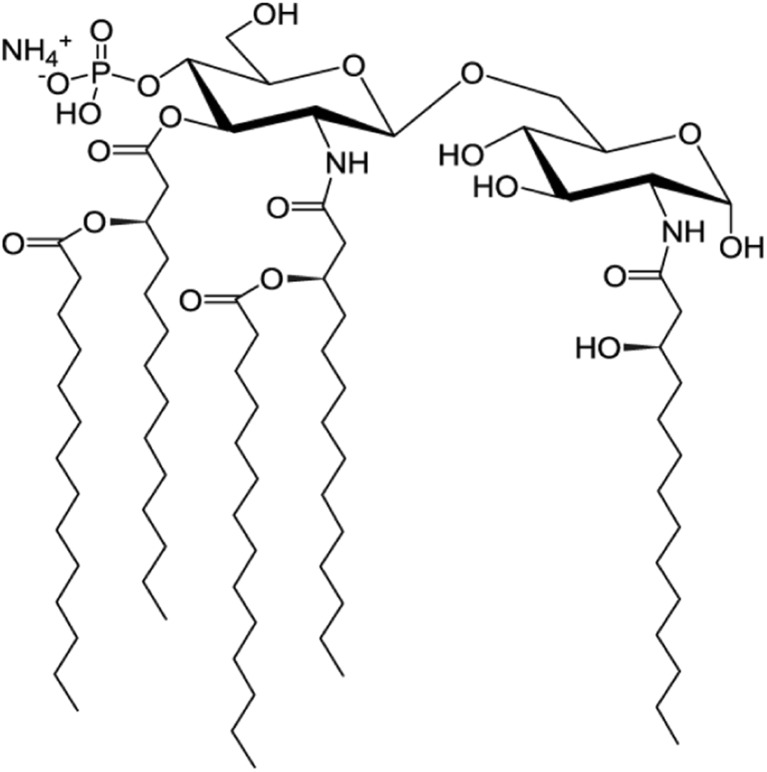
**3D-PHAD® molecular structure**. Phosphorylated HexaAcyl Disaccharide, a fully synthetic produced monophosphoryl lipid A (source illustration: https://avantilipids.com/product/699852; Accessed March 20, 2018).

**Fig. 2:**
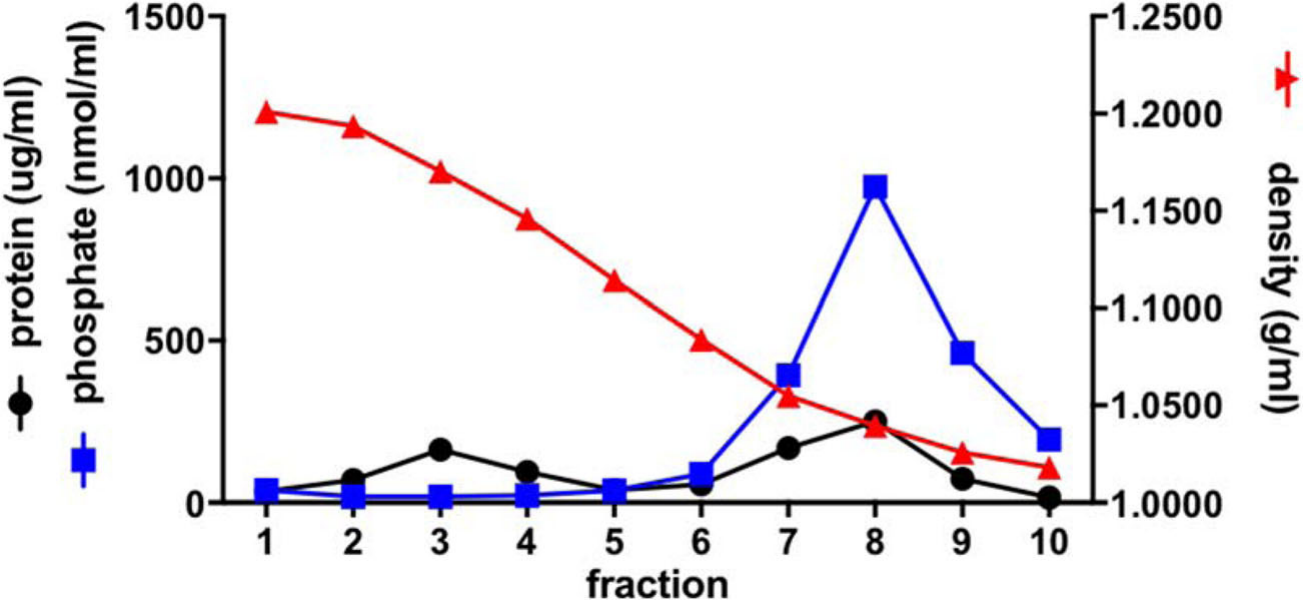
**Equilibrium density sucrose gradient analysis of virosomes.** An RSV A2 virosome preparation was analyzed by equilibrium density gradient centrifugation on a 10–60% sucrose gradient. Fractions of 0.5 ml were taken from the bottom (left side), and the density, protein and phosphate concentration of each fraction was determined.

The formation of virosomes was analyzed by equilibrium sucrose density-gradient centrifugation (Fig. 1). Figure 2 shows the results for a typical virosome preparation. Protein and phosphate were found to co-migrate in a single peak in the gradient, indicating successful reconstitution of the viral envelope. The absence of phosphate outside the virosome peak indicates that DOPC, DOPE and 3D-PHAD® (which all have phosphate groups) were essentially quantitatively associated with the virosomal membrane. Some non-incorporated protein was present in fraction 3 of the gradient.

**Fig. 3:**
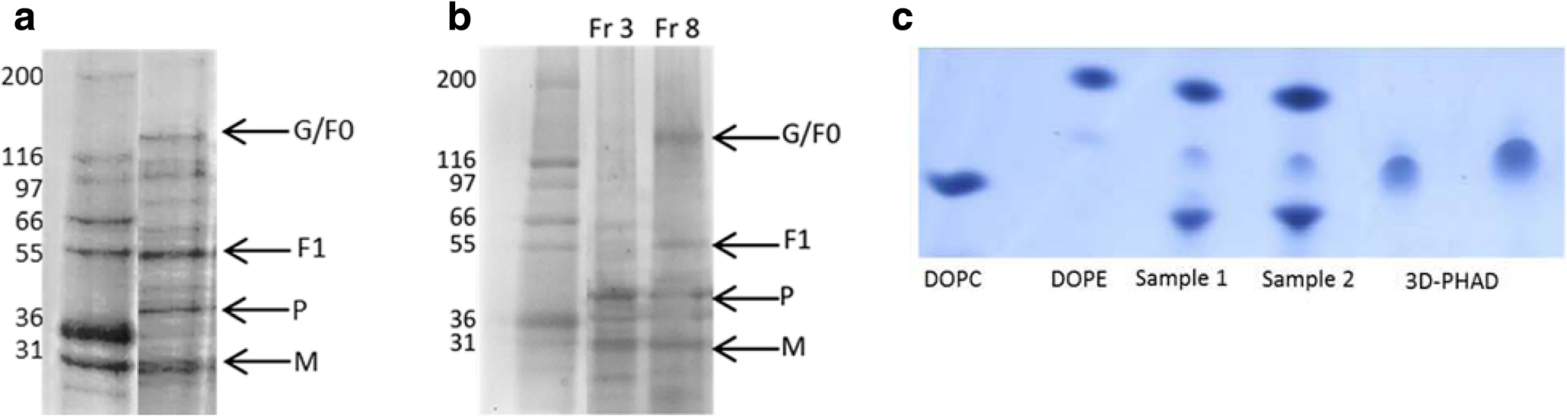
**Biochemical analysis of RSV virosomes.** (**a**) SDS PAGE analysis of virosomes. G: G glycoprotein, F0: inactive precursor F glycoprotein, F1: large subunit of F glycoprotein, P: nucleocapsid protein, and M: Matrix protein. (**b**) SDS-PAGE of fractions from the gradient in Fig. 1. (**c**) Virosomes were analyzed for DOPC, DOPE, 3D-PHAD® incorporation by thin layer chromatography (TLC), sample is present at two different dilutions (sample 1 and sample 2). Spot intensity measurements were done using ImageJ software.

The protein composition of the virosomes was analyzed by SDS-PAGE. In unfractionated virosome preparations, the F and G membrane proteins were found (Fig. 3a), along with the viral matrix (M), and phosphoprotein (P). G, uncleaved or non-reduced F (F0), and its F1 subunit were identified by blot (not shown); the F1 subunit has an apparent molecular weight of 54 kDa, and the G-protein was around 120 kDa in size, corresponding to the sizes of F and G reported in the literature (39–41). On the gel, the position of uncleaved F (F0) overlaps with that of G (Fig. 3a)**.** In the virosome fraction of the gradient, F, G and M were present. The non-incorporated protein consisted mainly of M and P, most likely from residual nucleocapsid (Fig. 3b). Semi-quantitative analysis of M and P band intensity of fraction 3 and 8 by ImageJ revealed that 46% of M and 40% of P are present in virosome fraction 8.

The incorporation of lipids and adjuvant into the virosomes was analyzed by thin-layer chromatography (TLC). The results of the TLC analysis confirmed that DOPC, DOPE and 3D-PHAD® were present in the virosome preparation (Fig. 3c). To roughly quantify the amount of incorporated lipids and adjuvant, the intensities of the spots observed after TLC analysis of the virosomal lipids, shown in Fig. 3c, were determined using ImageJ analysis. The relative recoveries of DOPE and DOPC in the virosomes were essentially equal and represented approximately half of the amounts that were initially added, indicating a somewhat lower recovery than that of the viral protein (64%). However, the total quantity of virosome-associated adjuvant 3D-PHAD® was found to be less than 10% of the amount initially added. It therefore appeared that 3D-PHAD® was specifically lost during the production process.

### Immunogold Labeling

The presence of F and G protein in the virosomal membrane, as shown in Fig. 3a, was further examined by double immunogold electron microscopy staining. With this technique, F and G were visualized simultaneously using specific monoclonal antibodies and secondary staining antibodies coupled to 6-nm gold particles for the F protein and to 15-nm particles for G protein. The double immunogold staining gives an indication for the ratio of F and G protein incorporated in the virosomal membrane compared to that in the native virus.

**Fig. 4:**
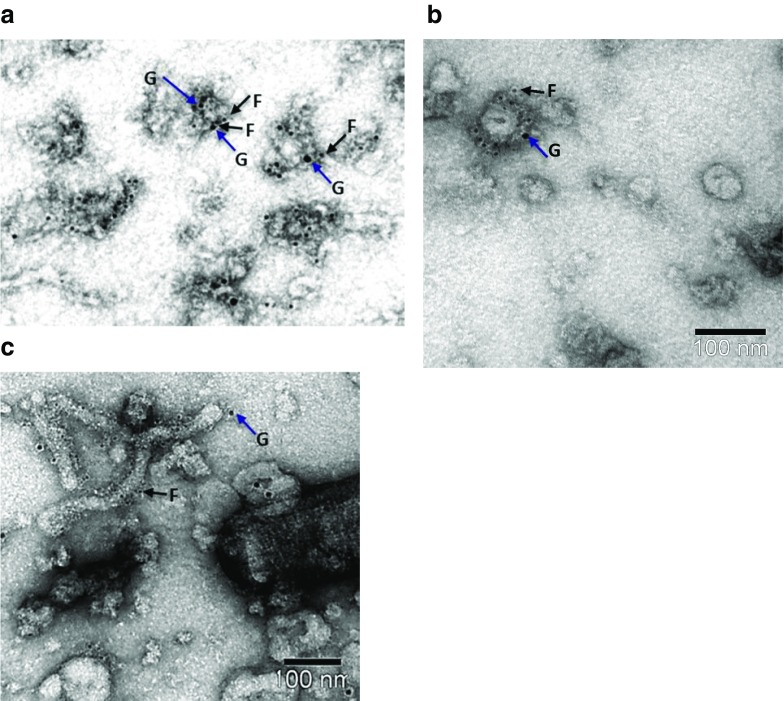
**Double immunogold labeling of virosomes and purified RSV.** Virosomes (**a**) and purified virus (**b** and **c**) were labeled with two different sizes of gold particles. The G protein was labeled with 15 nm gold particles and the F protein with 6 nm gold particles. Negative stain EM images, scale bars are 100 nm

Several experiments showed that, in different virosome samples, the amount of virosome-incorporated F protein was higher than that of G, in agreement with the F-to-G ratio in native RSV virions. Detailed examination of the electron micrographs showed that the F-to-G ratio in the virus sample shown in Fig. 4b**/**c was 7.1:1, while that in the virosomes shown in Fig. 4a was 5.3:1. Whereas the F-to-G ratio was thus similar in virus and virosomes, the overall membrane surface density of both envelope glycoproteins together appeared substantially lower in the virosomes than in the virus (Fig. 4)**.** This reduced density results from the presence of added lipids in the virosome membrane, which expands the membrane, and also from the fact that in virosomes, in contrast to virus, the membrane glycoproteins are randomly oriented, facing the inside of the virosome as well as the outside (21).

Control single immunogold staining was performed to exclude possible interference between the gold-conjugated monoclonal antibodies directed to F and G. The results showed that the number of gold particles bound to F or G did not vary between the single or double immunogold staining procedures (results not shown). This indicates that during double immunogold staining, the mAbs did not interfere with each other. Other controls (as described in Materials and Methods) showed that there was no cross-reactivity between the antibodies binding to F or G (results not shown).

### Size Distribution Analysis of the Virosomes

**Fig. 5:**
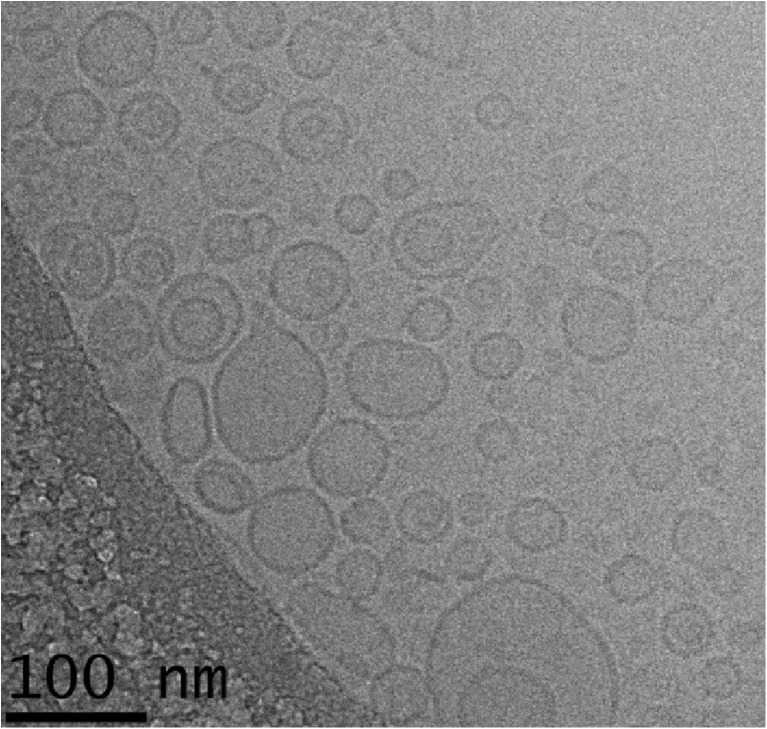
Cryo-electron micrograph of RSV virosomes.

**Fig. 6:**
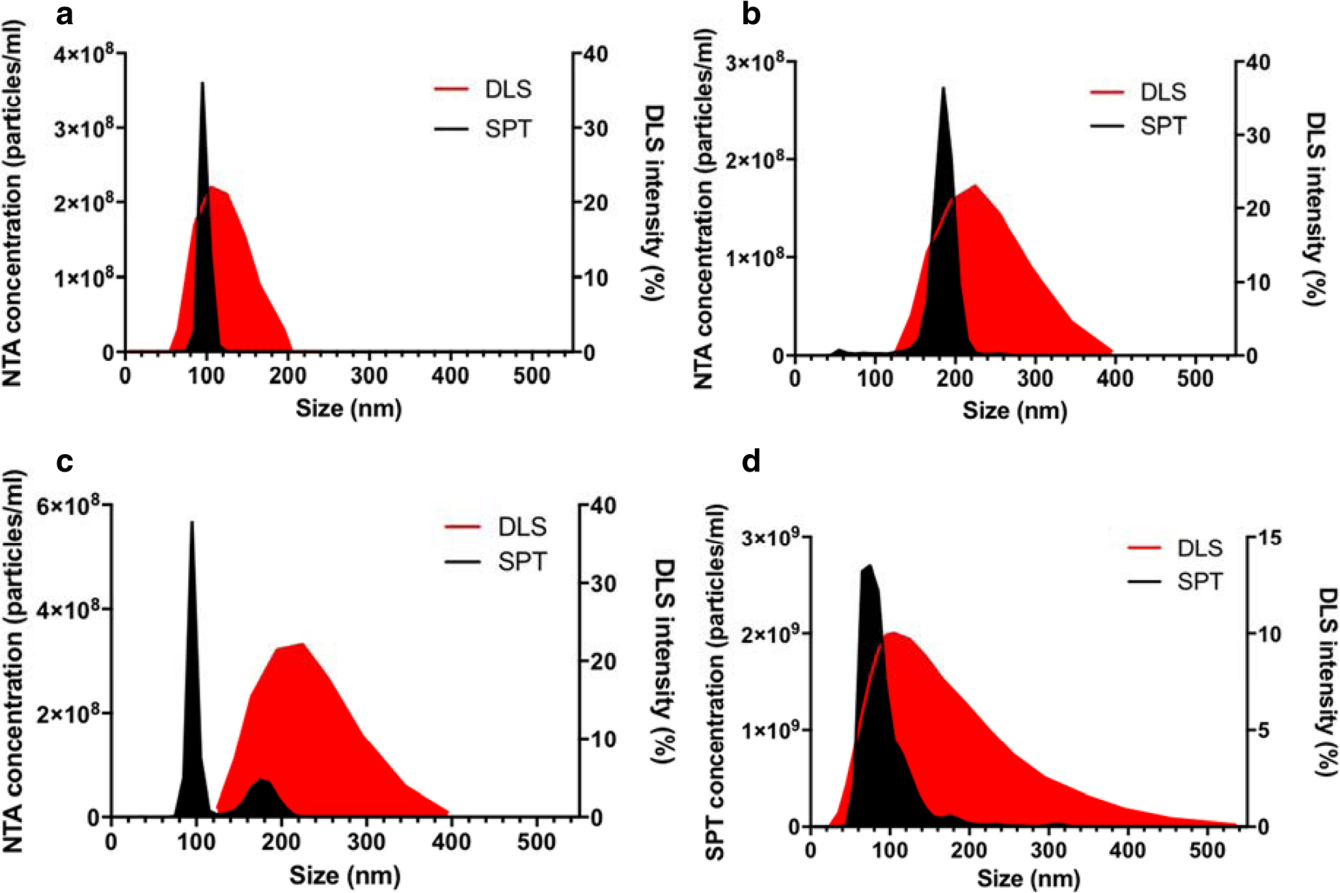
**Polystyrene beads in monodisperse and polydisperse suspension and virosome sample measured with SPT and DLS.** Panel (**a**), size distribution of 100 nm polystyrene beads, Panel (**b**) size distribution of 200 nm polystyrene beads, panel (**c**) mixture (2.5:1) of polystyrene beads of 100 nm and 200 nm and panel (**d**) Size distribution of virosome sample. Size distributions were measured by DLS (red) and SPT (black). Notice different scales.

The size distribution of virosomes is usually determined by dynamic light scattering (DLS) (42). By cryo-electron microscopy the virosomes appeared heterogeneous, and a large majority was smaller than 100 nm (Fig. 5)**.** However, by DLS it appeared as though the particles were much larger, with a broad size distribution, indicating a large fraction of particles with sizes between 200 and 500 nm (Fig. 6d). Such particles were not seen at all by microscopy.

To understand these discrepancies and find a reliable method to accurately determine the size of the virosomes, size measurements by single-particle tracking (SPT) were tested, and some model studies were undertaken. DLS is a population-based technique, which measures the mean hydrodynamic diameter of particles in suspension based on fluctuations in the light scattering produced by the particles (43–46). Particles are best described by the Z-average diameter, the intensity weighted harmonic mean using this technique, but that parameter may not accurately measure particle size in heterogeneous populations of particles. A Polydispersity Index (PdI) is therefore calculated from the data. The lower the PdI the more homogenous the suspension. For virosomes, a PdI of <0.4 is acceptable (42). Single-particle tracking (SPT) measures the Brownian motion of individual particles in liquid, which is directly related to the size of the particles by the Stokes-Einstein equation (47). For this technique, the best measure of size is the mode, the particle size that is most frequent in the population.

The reliability of size measurements in samples by DLS *vs*. SPT was first examined using monodisperse suspensions of polystyrene beads of 100 nm and 200 nm particle diameter, as well as a mixture of beads of 100 nm and 200 nm in a ratio of 2.5:1.

DLS and SPT measured similar Z-average diameters and modes, respectively, for either 100-nm (93 nm by SPT, 106 nm by DLS) and 200-nm (179 nm SPT, 212 nm DLS) polystyrene beads in suspension alone, showing a single peak of in the size distribution histogram (Fig. 6). Compared to SPT, the size distribution as measured by DLS was much broader, from 50 to 200 nm for the 100-nm beads, and 110–400 nm for the 200-nm beads. The polydispersity Index (PdI) of the DLS measurements was low, 0.026 for the 100-nm and 0.021 for the 200-nm beads. However, with the mixture of 100- and 200-nm beads, DLS did not resolve the two populations (Fig. 6c)**.** DLS showed one broad peak for the mixture, with a mean size of 207 nm, which is biased towards the larger particle size, although the majority of the particles was 100 nm. In spite of this, the calculated PdI was low also, 0.095, for the mixture of 100-nm and 200-nm beads, which would indicate a monodisperse sample. Therefore, the DLS data do not accurately represent the size distribution of the sample, without revealing the problem by an elevated PdI. In contrast, SPT clearly showed a large peak at around 100 nm (mode 96 nm) as well as a small peak at around 200 nm.

Figure 6d shows the size distribution of the virosomes measured by SPT and DLS. The SPT measurement showed a modal size of 96.3 nm. There was a clear right-hand shoulder in the size distribution, indicating a population of larger particles, or aggregates of smaller particles. The same virosome preparation measured by DLS showed a much broader peak with a Z-average of 113.1 nm and a PdI of 0.358. As for the mixture of polystyrene beads, DLS measurements of the virosomes thus showed larger mean particle diameters than SPT and a broader size distribution, which could be caused by the larger particles or aggregates revealed by SPT dominating the signal in DLS. By SPT, D10, D50 and D90 values for the virosome preparation were 52.8 nm, 75.3 nm and 107.1 nm. The D50 value, the median, is defined such that half of the particle population has a diameter smaller than this value. Likewise, 90% of the particles have a diameter below the D90 value, and 10% below the D10 value. It was concluded that SPT measurements were much more representative of the virosomes as observed by electron microscopy (Fig. 5)**,** with the exception of the right-hand shoulder; by EM, particles larger than 100 nm were not observed. It is possible that in solution, some virosomes would aggregate and move as one large particle. By EM that cannot be verified.

### Stability and Aggregation of RSV Virosomes

**Fig. 7:**
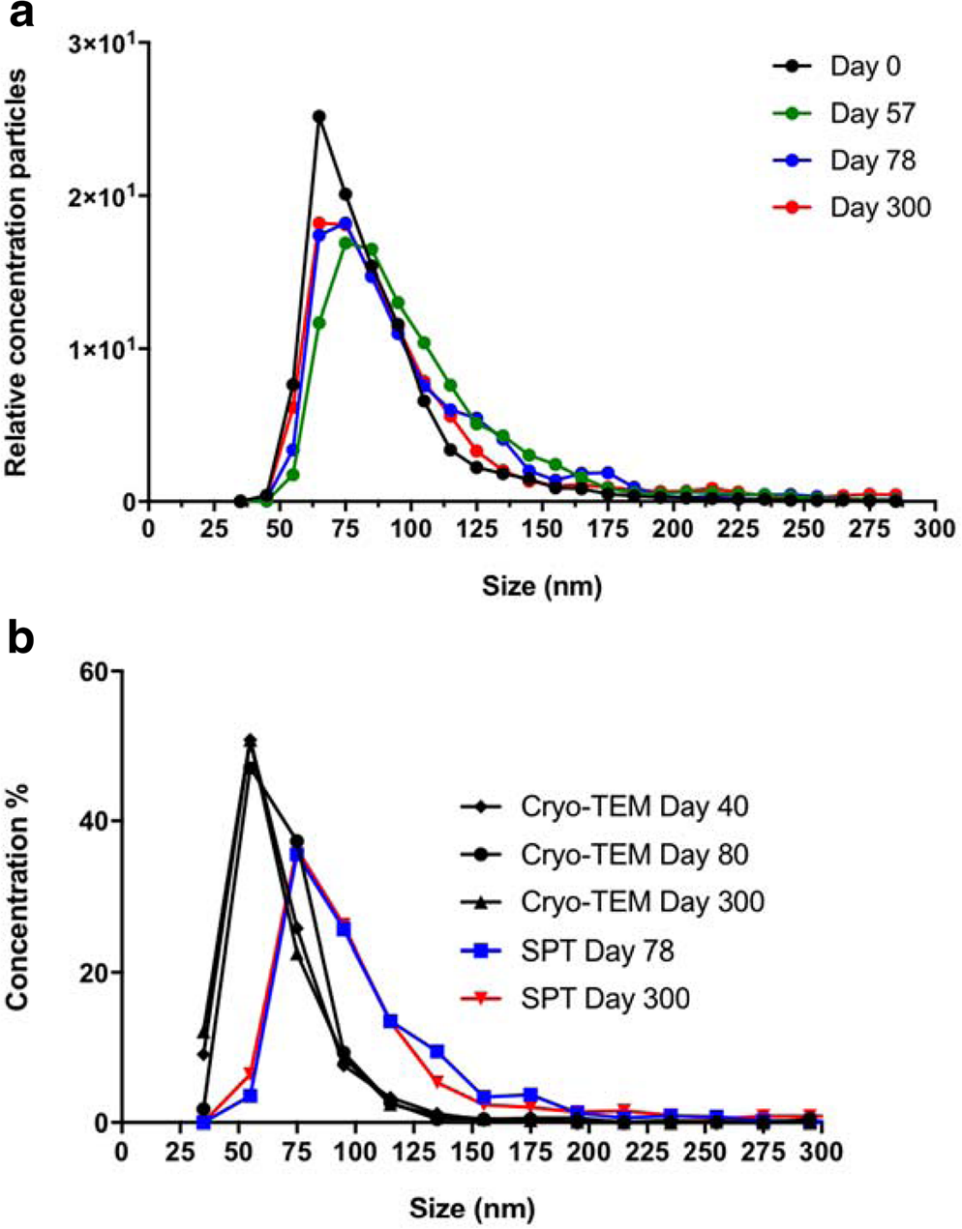
**SPT and Cryo-TEM size distributions of RSV virosomes.** Panel (**a**) Size distribution measured by SPT. Each time point is the average modal size of at least six individual measurements, each representing at least 10,000 finite tracks (about 1500 particles). Panel (**b**) Average diameter of particles as measured by cryo-electron-microscopy, 330 particles were measured for the 40-day time point, 225 for the 80-day, and 1597 for the 300-day point. For comparison, some of the corresponding SPT measurements are shown.

Virosome samples were analyzed over time for their long-term stability to investigate if aggregates were accumulating. A fresh preparation of virosomes was measured immediately after production and then stored at 4°C for 300 days. Periodically, samples were taken and the size distribution was measured with SPT (Fig. 7**,** Table I) and cryo-TEM (Table II). It was observed that the virosomes would sediment over time, potentially indicating aggregation of the particles. Before each SPT measurement, or electron microscopy, samples were inverted several times to resuspend the sediment, and diluted with buffer. As measured by SPT, the mode and D10 changed very little over time. However, between freshly produced virosomes and all other time points, there was a slight increase in D90, and in the number of particles in the right-hand shoulder of the distribution. An arbitrary cut-off of 150 nm was chosen to characterize these (Table I). The size of the particles as measured by EM was consistently smaller than that measured by SPT and showed no trend over time. The difference was not due to the size limit in SPT; SPT cannot measure particles smaller than 30 nm, but by EM, such particles were not detected.

**Table I:** Size Distribution of Virosomes Measured by SPT Over a Period of 300 Days

Date	Day 0	Day 57	Day 78	Day 300
% <150 nm	97%	93%	91%	90%
% >150 nm	3%	7%	9%	10%
Mode (nm)	69	82.4	75.3	72.4
D10 (nm)	50.7	57.4	53.8	52.1
D90 (nm)	110.9	142.4	140.3	155.2

For each time point, the reported parameters were the average of at least 6 individual particle size distribution measurements, each representing about 10,000 complete particle tracks

**Table II:** Size Distribution of Virosomes Measured by Cryo-TEM Over a Period of 300 Days

	Day 40	Day 80	Day 300
Average (nm)	57.7	60.5	55.3
Mode (nm)	48.3	73.3	48.3
Particle count	330	225	1597

### Improved Incorporation of 3D-PHAD® in the Virosomes

As indicated above, the recovery of the 3D-PHAD® adjuvant in the virosomes was significantly lower than that of the other virosomal components (Fig. 2). In the initial virosome production process, the lipids were deposited as a thin lipid film on the wall of a glass tube, which was then dissolved in the viral supernatant, which contained 100 mM of the short-chain phospholipid DCPC, acting as a detergent (its critical micelle concentration is about 17 mM). The 3D-PHAD®/viral protein ratio in the mixture was 1 mg/mg. The resulting mixture was clear, suggesting all components were dissolved. After sterile filtration, virosome were formed from the filtrate by dialysis.

In order to investigate the loss, and to measure 3D-PHAD® more precisely, two contract research organizations independently developed methods to measure its concentration in a virosome sample by LC-MS, reaching similar conclusions (Table III); indeed, less than 1% of the added adjuvant was incorporated in the virosomes. Heating the glass tube with the lipid film/viral supernatant mixture to about 45°C for about 30 min, increased the virosome-incorporated amount of 3D-PHAD® tenfold. It was next attempted to dissolve 3D-PHAD® in 100 mM DCPC and add it to the viral supernatant. Although the solution appeared clear, after filtration the resulting virosomes still contained less than 10% of the initial amount of 3D-PHAD®. Subsequently, 1 mg/ml of 3D-PHAD® was dissolved in 500 mM DCPC (the limit of DCPC solubility in water), with bath sonication for 5–8 min at 56°C, and mixed with stock solutions of the lipids, also in DCPC. The mixture was added to the viral supernatant, and virosomes were prepared without filtration. At these concentrations of DCPC, it took 48 h with seven changes of buffer, to completely dialyze out the DCPC. The 3D-PHAD® was incorporated quantitatively (Table III). This was then repeated with sterile filtration of the mixture. Apparently, filtration led to the loss of viral protein, but all the 3D-PHAD® was incorporated in the virosomes, leading to an elevated 3D-PHAD® over protein ratio (Table III).

**Table III:** 3D-PHAD® Incorporation in Virosomes

Preparation	3D-PHAD® incorporated (mg/mg of protein)
Virosomes from a dry film of lipids and 3D-PHAD®, filtration	0.009
Virosomes from a dry film of lipids and 3D-PHAD**®**, heated, filtration	0.09
Virosomes from a dry film of lipids, 3D-PHAD**®** solubilized @ 100 mM DCPC, filtration	0.08
Virosomes from solubilized lipids @ 200 mM and 3D-PHAD**®** @ 500 mM, without filtration	0.995
Virosomes from solubilized lipids and 3D-PHAD**®** @ 500 mM, filtration	1.5

In all cases, 1 mg of 3D-PHAD**®** was added to 1 mg of viral protein during the production of virosomes

**Fig. 8:**
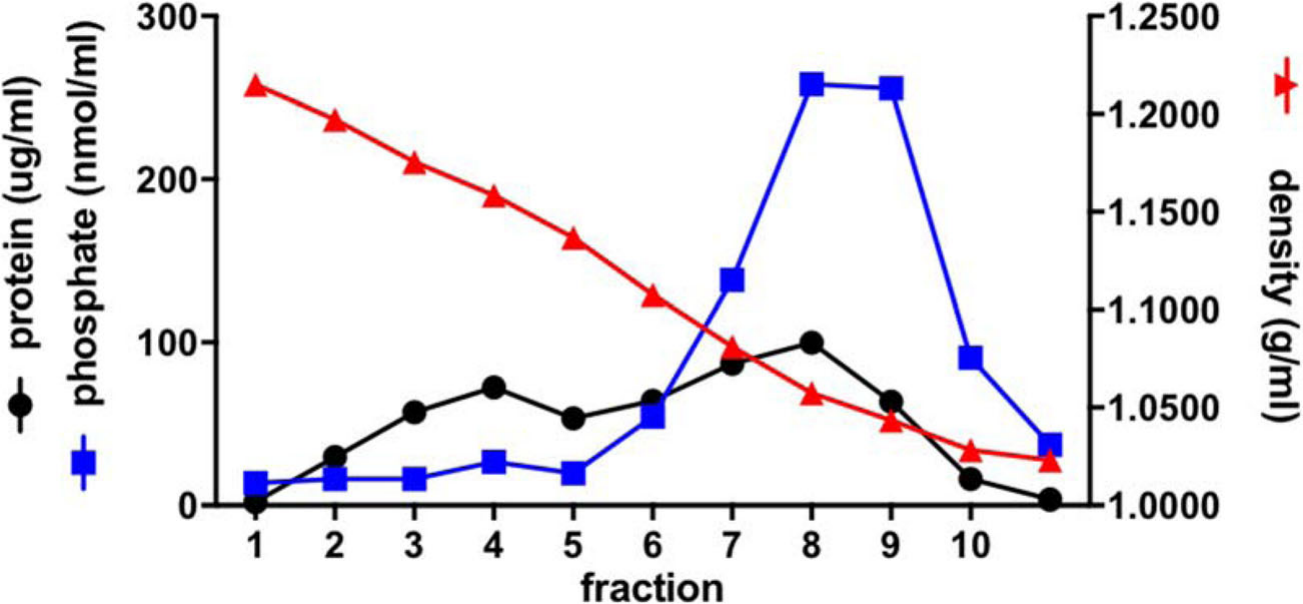
**Equilibrium density sucrose gradient analysis of virosomes.** After adding from DMSO to virosomes and overnight incubation, virosomes were analyzed by equilibrium density gradient centrifugation on a 10–60% sucrose gradient. Fractions of 0.5 ml were taken from the bottom (left side), and the density, protein and phosphate concentration of each fraction were determined.

Although it was thus possible to incorporate 3D-PHAD® quantitatively in virosomes, the final concentrations of DCPC in the protein/lipid/adjuvant mixture were more than 250 mM, prolonging dialysis times. It was therefore attempted to incorporate 3D-PHAD® in pre-formed virosomes from a solution of 10 mg/ml of 3D-PHAD® in DMSO. Virosomes were prepared without 3D-PHAD®, and a small aliquot of 3D-PHAD® in DMSO (25 μl) was slowly added to 975 μl of virosomes with constant stirring at room temperature, at a ratio of 0.1 mg of 3D-PHAD® per mg of viral protein, and incubated overnight at 4°C. The virosomes were then analyzed by sucrose density gradient centrifugation (Fig. 8). As in Fig. 2, a single peak of phosphate was found for the virosomes. The 3D-PHAD® was found to be quantitatively incorporated in the virosomes.

### Immunogenicity in Mice of RSV Virosomes with Improved Levels of 3D-PHAD®

**Fig. 9:**
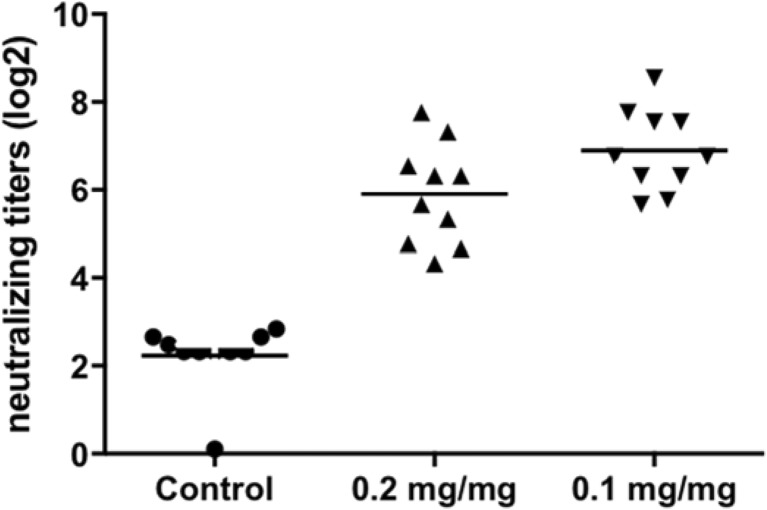
**RSV-specific neutralizing antibodies.** Mice (10 animals per group) were vaccinated twice IM with RSV virosomes containing 3D-PHAD®. Control groups were vaccinated with buffer. Each injection contained 5 μg of protein. Group 0.2 mg/mg represents 0.2 mg/mg 3D-PHAD®/viral protein in virosomes prepared by the classical, thin lipid film methodology RSV. Group 0.1 mg/mg represents 0.1 mg 3D-PHAD®/viral protein post-inserted after virosome preparation. Neutralizing antibody titers in serum obtained at day 28. Logarithmic (base 2) representation of the titer, the line denoting the average.

**Fig. 10:**
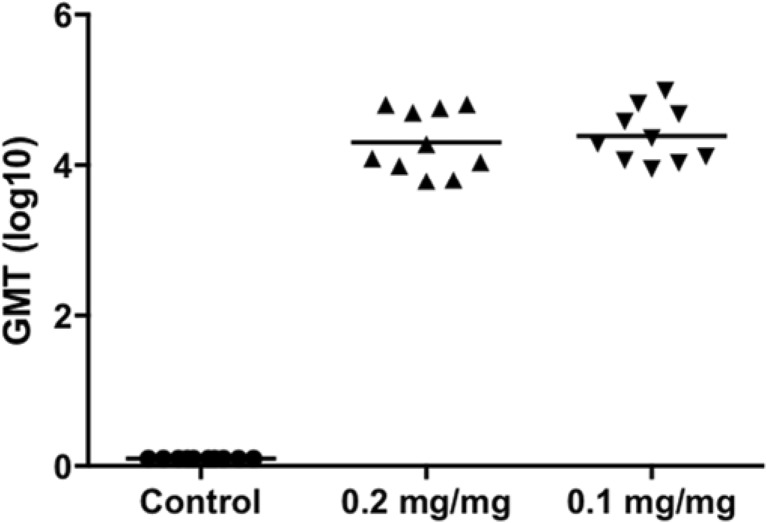
**Anti-RSV IgG as determined by ELISA.** Mice (10 animals per group) were vaccinated twice IM with RSV virosomes containing 3D-PHAD®. Control groups were vaccinated with buffer. Each injection contained 5 μg of protein. Group 0.2 mg/mg represents 0.2 mg/mg 3D-PHAD®/viral protein in virosomes prepared by the classical, thin lipid film methodology RSV. Group 0.1 mg/mg represents 0.1 mg 3D-PHAD®/viral protein post-inserted after virosome preparation. Logarithmic (base 10) representation of the geometric mean titer, the line denoting the average

To evaluate the immunogenicity of RSV virosomes with improved incorporation of 3D-PHAD® and to compare virosomes prepared by the DMSO method and the DCPC method, an immunization experiment in mice was performed. Mice were immunized twice, on day 1 and day 15 with virosomes, prepared by the DMSO method at 5 μg of protein per injection. In the comparator group, mice were immunized with virosomes containing 0.2 mg 3D-PHAD® per mg of protein, with 3D-PHAD® dissolved in DCPC, as described above, reasoning that the 3D-PHAD® added from DMSO would only be present in the outer leaflet of the virosomal bilayer, and that only 3D-PHAD® presented to the immune system on the outside of virosomes would act as an adjuvant. Blood samples were taken at day 28 and analyzed for neutralizing antibodies (Fig. 9) and anti-RSV IgG (Fig. 10) as described before (33). Both preparations were found to induce similar antibody titers, indicating the quality of the virosomal vaccines produced by either method is equivalent.

## Discussion

In earlier studies, an MPLA-containing virosomal RSV vaccine was tested in mice and cotton rats, and found to induce a good immune response with a favorable safety profile (33,34,36). Those virosomes were made by dissolving the viral membrane in DCPC, removing the nucleocapsid, adding the dissolved membranes to a dry lipid film of lipids containing MPLA, followed by sterile filtration and then dialysis of the DCPC. However, MPLA is a complex mixture of molecules which is difficult to produce and to reproduce. Aiming to produce virosomes under GMP, in the current paper, a synthetic variant of MPLA, 3D-PHAD®, was incorporated in the virosomal membrane and the virosomes were analyzed extensively. As expected, the viral membrane proteins F and G were found in the virosomes, in a ratio similar to that of the virus, albeit at a lower surface density than on virions. The lower surface density resulted from the expanded membrane, as lipids were added during virosome formation to the solubilized viral membrane components, and because of the random orientation of the membrane proteins in the virosomal membrane as opposed to the virus, where they face outward only. The lipid addition serves mainly to accommodate the adjuvant in the virosomal membrane. If the virosomes are not further purified, the preparation additionally contains at least the viral proteins P and M. About half the M protein was found to be virosome-associated after sucrose density gradient purification; the rest of the M and P were not virosome-associated. Most likely, associated M is bound to the C-terminus of F, as M drives the budding of virus by associating with F (48). While virosomes, which were made using a mixed dry lipid film of DOPE, DOPC and 3D-PHAD®, contained approximately 50% of the added lipids, and all 3D-PHAD® was found in the peak of the gradient (not shown), only a fraction of the 3D-PHAD® appeared to be virosome-incorporated.

These virosomes were then further characterized by size distribution measurements. Although the standard method for virosome size measurements is DLS, DLS showed much larger virosomes than could be seen by cryo-electron microscopy. When virosomes were measured by SPT, the size distribution partially overlapped with diameters determined from cryo-electron micrographs, except for two features; EM showed more particles with a diameter between 30 and 50 nm, while SPT showed relatively more particles of 100–125 nm, and the size distribution by SPT had a right-hand tail, showing a fraction of particles with sizes between 150 and 200 nm (Fig. 7b). These data suggest that small particles might aggregate in solution, with small aggregate movements jointly producing a single track in SPT measurements. Model studies were then undertaken with polystyrene beads. These studies showed that a 2.5:1 mixture of 100- to 200-nm beads is resolved by SPT (Fig. 6c), but produces essentially a single, broad size distribution around the size of the larger particles if measured by DLS, just as the distribution found for the virosomes by DLS (Fig. 6d). Therefore, we conclude that small aggregates, whose presence is suggested by the SPT measurement, are causing the scattering giving rise to the distribution measured by DLS. As an indication for the underlying polydispersity of a sample, DLS instruments calculate a PdI. A PdI of <0.7 is usually taken to mean that polydispersity is low, and the size distribution measurement reliable (49). However, both virosomes and the latex beads mixture have a polydispersity index lower than 0.7.

We noticed that virosomes would sediment after weeks to months of storage in the refrigerator, suggesting they may have a tendency to agglutinate or aggregate. Irreversible aggregation would not be acceptable within the context of production under the regulations of Good Manufacturing Practice (GMP) (50,51). Also, particle size can have a substantial influence on the immunogenicity of vaccines. Therefore, the size distribution of the virosomes was followed for a prolonged period of ten months. Virosomes had to be resuspended before each measurement. Over this period, the modal size and D10 remained unchanged, while there was an only slight increase in D90 and the number of particles >150 nm between production and the first time-point, 8 weeks later. After that, the size distribution remained constant. We conclude that the virosomes are stable and that aggregation, if it occurs al all, is largely reversible. It is possible that upon long-term storage the particles do not even agglutinate, but simply sediment because of their higher density relative to that of the medium.

Although the optimal concentration of 3D-PHAD® in virosomes is not currently known pending further animal experiments, more control over the extent of 3D-PHAD® incorporation was desired. 3D-PHAD® is less soluble than phospholipids in organic solvents, with a solubility limit of around 1 mg/ml in chloroform/methanol 2:1, suggesting it is more amphiphilic. However, since DOPC and DOPE were incorporated into virosomes from a dry lipid film also containing 3D-PHAD®, while the adjuvant was not, the solubility of 3D-PHAD® in DCPC seemed limited. A solution of 3D-PHAD® in 100 mM of DCPC appeared clear, but when added to the viral supernatant and filtered, it did not lead to more incorporation. We suspect that the filtration removed aggregates or micelles of 3D-PHAD® which were not visible to the naked eye. 3D-PHAD® in 500 mM DCPC, dissolved by heating and sonication and immediately added to the viral supernatant, was incorporated quantitatively, and this 3D-PHAD®/DCPC mixture could be filtered, either mixed with the viral supernatant (Table III) or on its own (not shown). However, this is not the most convenient production method, as the high concentrations of DCPC may damage proteins and lead to long dialysis times, and low temperatures are preferred. Finally, virosomes without adjuvant were formed first, and then 3D-PHAD®, which dissolves in DMSO at up to 10 mg/ml, was added from DMSO. As the DMSO mixes with water, 3D-PHAD® becomes insoluble and inserts into the virosomal membrane; the sugar residues most likely prevent translocation across the membrane, so that it is only present in the outer leaflet of the virosomal membrane. In accordance with this, an equivalent immune response was obtained with virosomes containing 0.2 mg/mg 3D-PHAD®/protein in both leaflets of the membrane, and 0.1 mg/mg after post-formation insertion from DMSO (Figs. 9, 10). An added advantage of post-formation insertion of 3D-PHAD® is that the amount of adjuvant per virosome can be halved.

## Abbreviations

3D-PHAD^®^: 3-deacyl-phosphorylated hexa-acyl disaccharide
Cryo-TEM: Cryogenic-transmission electron microspcopy
DCPC: 1,2 dihexanoyl-*sn*-glycero-3-phosphocholine
DLS: Dynamic light scattering
DOPC: 1,2-dioleoyl-*sn*-glycero-3-phosphatidylcholine
DOPE: 1,2-dioleoyl-*sn*-glycero-3-phosphatidylethanolamine
FI-RSV: Formalin inactivated-RSV
F protein: Fusion glycoprotein
GMP: Good manufacturing process
G protein: Attachment glycoprotein
HPLC: High-performance liquid chromatography
LC-MS: Liquid chromatography–mass spectrometry
MPLA: Monophosphoryl lipid A
PdI: Polydisperse index
RSV: Respiratory syncytial virus
SARI: Severe acute respiratory infection
SPT: Single particle tracking
TLC: Thin layer chromatography
TLR4: Toll-like receptor 4
VN: Virus neutralizing
